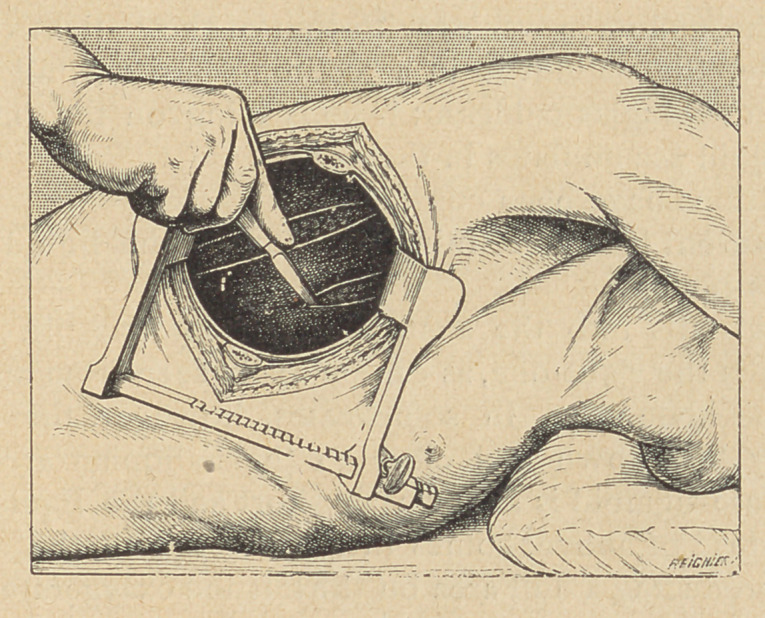# Treatment of Empyemata

**Published:** 1919-01

**Authors:** 


**Treatment of Empyemata.** By TH. TUFFIER. *La Presse Médicale.* September 26, 1918.

The author describes fully in this paper the treatment given by himself and by Dr. Depage in cases of " medical " purulent pleurisy and of surgical post-traumatic empyemata.

*Treatment of Closed Pleural Suppurations.*

This treatment comprises : the pleurotomy, the chemical disinfection, and the suture of the wound.

(1) *Pleurotomy.* A simple pleurotomy through the intercostal space should be resorted to if the purulent pleurisy is due to some pneumococcic infection. The incision is practised in the most sloping region, on a level with the posterior axillary line. The retractor being introduced into the wound, the pleura appears widely opened, and the complete discharge of the pus and false membranes is easily obtained.

If the case is not one of pneumococcic infection, a thoracotomy with resection of a single rib is advisable. It allows of the complete discharge of all pathologic exudates, of the wide retraction of the ribs, and of the examination "de visu" of the whole pleural cavity and of the lung.

This examination is most important to determine the exact size, the limits, and disposition of the suppurating focus, which sometimes presents deep and irregular recesses. The careful examination of the lung is also very necessary, as shown by the following instance: On examining the cavity in a case of purulent pleurisy, the author found a very thick and rough place in the pleuro-pulmonar wall; on incising that part of the pleural wall, and performing a complete decortication, he discovered a focus of abundant pulmonary effusion, wholly unsuspected and separated from the pleural cavity.

Finally Carrel tubes are inserted into the pleural cavity for its complete disinfection. Seven or eight tubes are inserted for that purpose in all recesses, and all directions, and fixed to the skin by adhesive plaster. In some cases a silver wire is introduced into the tubes, thus giving them a certain rigidity and helping to keep them in place.

(2) *Chemical Disinfection.* Dakin's solution is injected through each tube every two hours. Every two days samples of the effusion are collected on the skin, in the track of the wound and in the remotest recesses. Bacteriological examinations are made in order to determine the nature and the quantity of the infective germs.

After from 4 to 30 days, the pleural cavity is sterilized, as shown by the microbiological curve, and the research of the pathological germs.

(3) *Suture.* As soon as the sterilization is accomplished, the surgical incision is closed, according to the technic described further on. Great care must be taken that no blood effusion remains in the sterile cavity. Suitable breathing exercises are practised in order to favor the rapid absorption of the closed pneumothorax.

### *Treatment of Empyemata with Fistulae.*

In some cases a fistula remains after the operative treatment of purulent pleurisy.

Generally when the patient comes for reexamination the suppuration has existed for a period of from 6 weeks to one year, or more; the drainage orifice is small; the surgical incision partly closed; and the general condition rather poor.

The microbiological examination is practised by collecting samples of the exudate in the deepest part of the cavity, at the edges of the fistula, and on the surrounding skin :

The subsequent treatment consists of :

(1) Incision of the pleural adhesions, if necessary.

(2) Chemical disinfection.

(3) Suture of the wound.

(1) Several rubber tubes with inner silver wires are introduced into the cavity; this is then radiographed to determine the size and general shape of the cavity. If narrow and remote recesses exist, an injection of Bismuth proves useful before the X-ray examination. This examination is only practised in cases where the complete opening of the fistula is not resorted to immediately.

In the reverse case, the wound is opened widely in the corresponding intercostal space. On careful examination grayish pellicles are often found on the surface of the lung, which can be removed by rubbing gently. The condition of the surface, the disposition, number, and size of the diverticula of the false membranes require special attention. The cavity being infected, and the danger of sepsis always present, the only thing to do at first is to insert Carrel tubes within the wound.

If the false membrane comes off easily, it should be removed immediately. If the cavity proves sterile it should be closed without further delay.

(2) Dakin's solution is generally used, but it is ill supported, and occasions fits of coughing in cases of bronchial fistulae. In such cases oxygen can be resorted to every hour. While the process of disinfection is going on, special, methodical breathing must take place every day. The size of the pleural cavity is measured exactly by injecting into it a certain amount of fluid. The expansion of the lung is easily determined by the difference of the quantities injected during inspiration and expiration.

To make quite sure that the sterility of the pleura is complete, it is safe to do some bacteriological examinations 24 hours after the removal of the Carrel tubes and the suppression of the disinfecting treatment.

(3) The complete resection of the track of the fistula must first take place, with partial or total decortication, the thoracic wall being retracted as far as possible. The false membrane, resembling a true " pulmonary shell ", is cut through at the junction of the lung and the thoracic wall.

This part of the operation often proves rather difficult, a wide opening of the thoracic wall rendering it more easy, especially at the apex of the lung. The false membrane being separated as completely as possible with the knife's blade, a sort of long spatula is introduced between the lung and the membrane, and the pulmonary decortication resorted to. In some cases this can be complete, the lung expanding then from its " shell " and filling up the thoracic cavity.

This decortication aims at separating completely the lung and its thin pleura from the false membrane, the surface of the lung remaining untouched. A slight oozing of blood, and a few bubbles of air may occur when the parenchyma of the lung has been slightly injured.

When the total decortication proves impossible, all fragments of the false membrane that can be removed without seriously injuring the lung must be cut off.

If very intimate adhesions are met with, as is often the case around the primitive lesion, they must generally be left untouched, since their dissection inside the lung might prove dangerous.

If a bronchial fistula exists, it must be closed by " enfouissement à la Lambert ".

If the false membrane of the pleuro-parietal wall is not easily removed, it can be left behind without great damage.

If no blood oozes on compressing slightly the surface of the lung, after the operation, the flaps are mobilized, and the whole wound is sutured.

If the blood oozes, an incomplete suture must be resorted to. A light gauze dressing is placed upon the surface of the lung, which permits the absorption of the blood, and the wound is closed partially. On the next day the dressing is removed, the expectatory threads are tightened, and the suture completed.

This method of decortication is nothing more than " Delorme's operation ". Formerly its results were generally bad because the operation was performed in a septic medium, and a new empyema developed, together with a new false membrane.

The dangers of sepsis being removed, the gradual absorption of the pneumothorax is complete, and the expansion of the lung quite normal again.

The "pulmonary shell" being often infected throughout its depth, Prof. Tuffier thinks that still better results might be obtained by removing the membrane before the complete sterilization of the cavity.

The only post-operatory complications met with by the author were a second septic effusion, in general not very extensive, which had to be removed and sterilized, and a re-opening of the superficial region of the scar, due to some osteitis in the resected extremity of a rib. Delayed healing was the only consequence of these complications.

The actual tendency in this sort of operation is just the reverse of what it was formerly; the lung is made to expand towards the thoracic wall, and, thanks to this technic, the restoration of the normal function of the lung is complete.

Until June 1, the author had operated 47 cases:

(1) 7 medical empyemata.

(2) 40 empyemata following wounds in the lungs. Among these 40 cases, 3 were recent, 37 were chronic; all healed.

(1) Medical Cases. When the operation was performed, the empyema had developed

Twice in a fortnight.

Once in a month and a half.

Once in 2 months and a half.

Twice in 4 months.

Once in 3 years and a half.

The sterilization needed

Twice, 10 days.

Twice, 17 days.

Twice, a month.

Once, 4 months.

The wound was closed

Twice, 10 days after operating.

Twice, 17 days after operating.

Twice, a month after operating.

(2) Empyemata Following Wounds in the Lungs.

When Prof. Tuffier examined those cases, the empyema lasted from 20 days to one year..

The process of sterilization needed from a fortnight to 6 months

(a little more than a month in 15 out of 40 cases). In one case the cavity proved sterile and could be sutured immediately.

The wound was closed in lapses of time ranging from a fortnight to 9 months after the operation.

*Complications.* In 11 cases the wound had to be re-opened for suppurating effusion. In 2 cases the wound had to be re-opened twice. All these cases healed.

Two other cases had to be re-opened; one for a bony fistula, and one for hemoptysis.

A slight thoracic deformation was mentioned in 9 cases, and a very marked one in one case.

## Figures and Tables

**Figure f1:**
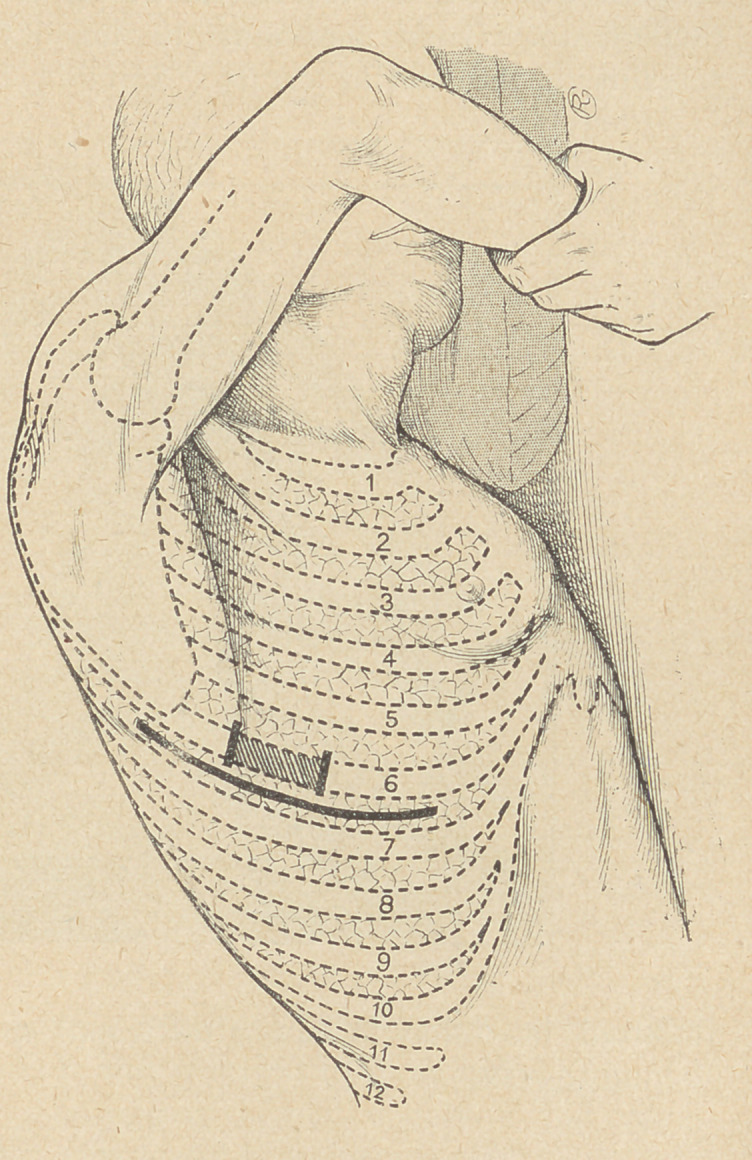


**Figure f2:**
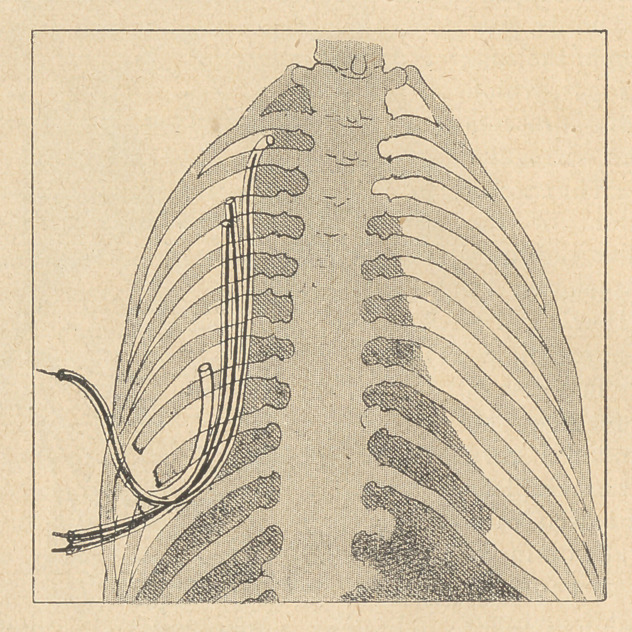


**Figure f3:**
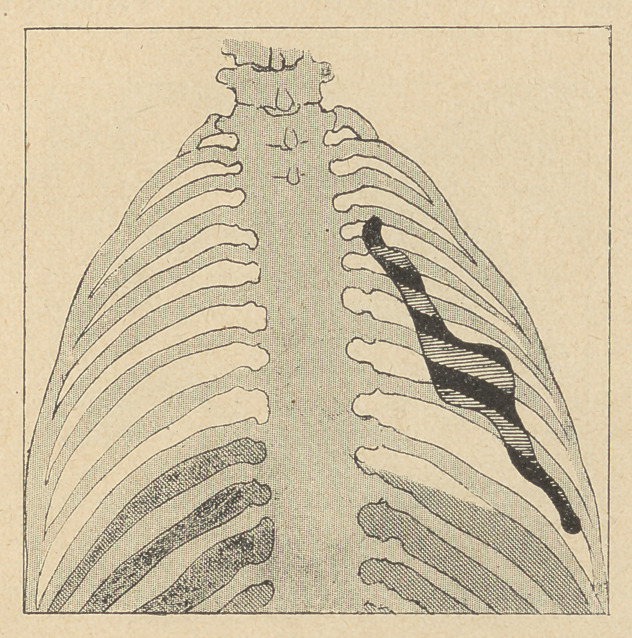


**Figure f4:**
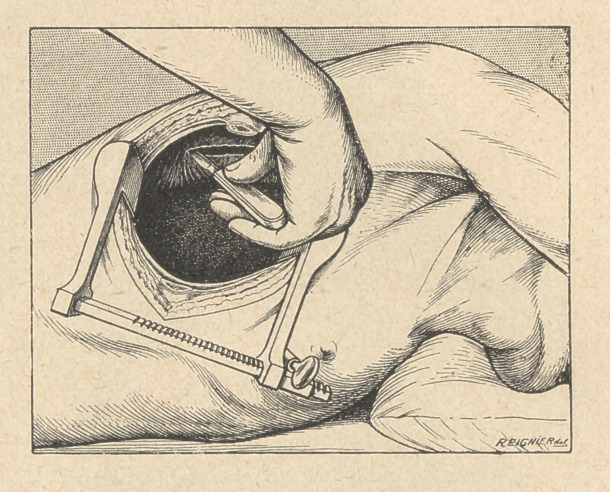


**Figure f5:**
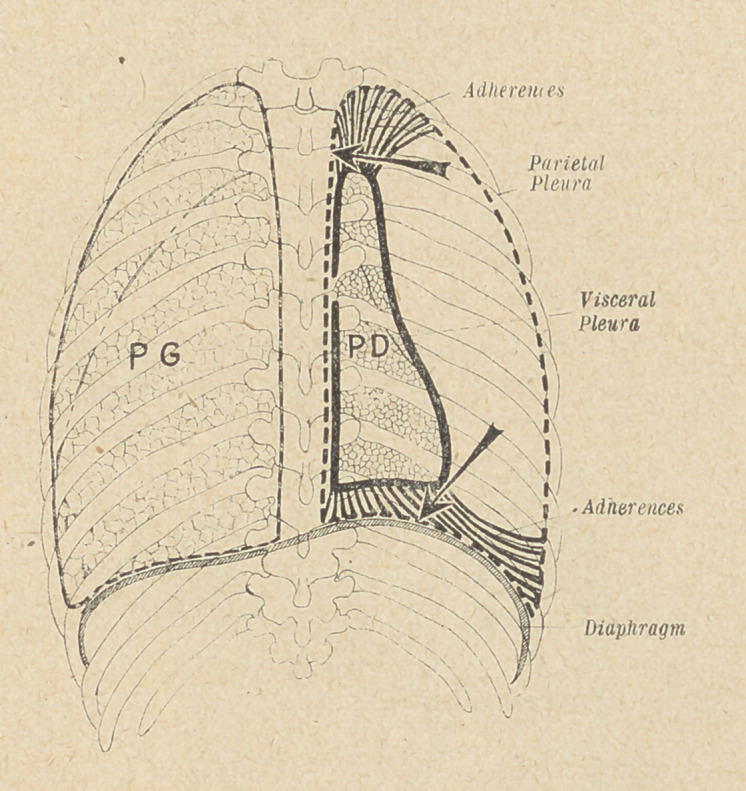


**Figure f6:**
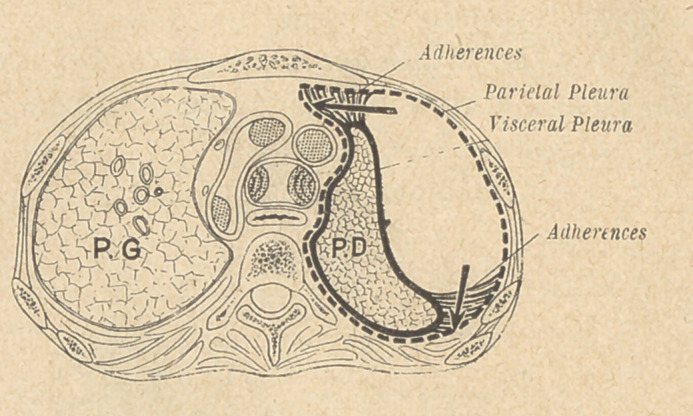


**Figure f7:**
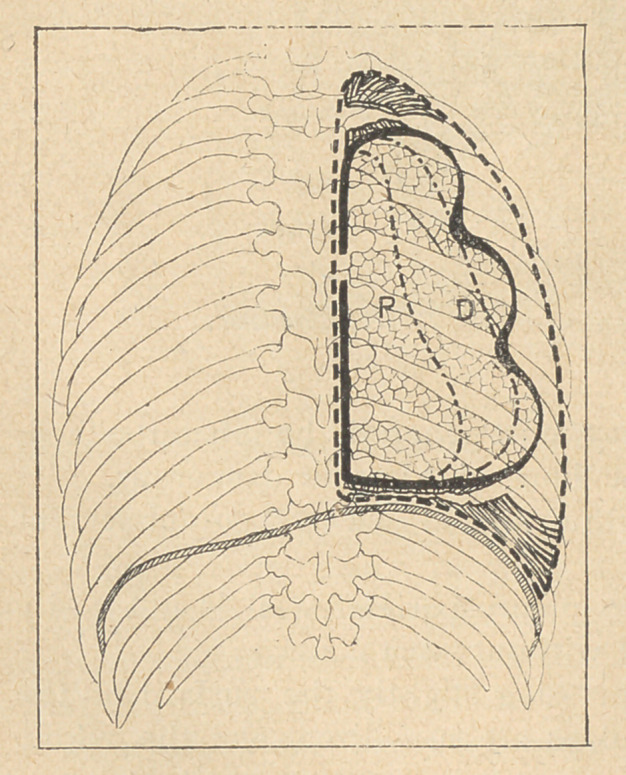


**Figure f8:**